# Maternal sensitivity and social support protect against childhood atopic dermatitis

**DOI:** 10.1186/s13223-017-0199-4

**Published:** 2017-05-26

**Authors:** Nicole L. Letourneau, Anita L. Kozyrskyj, Nela Cosic, Henry N. Ntanda, Lubna Anis, Martha J. Hart, Tavis S. Campbell, Gerald F. Giesbrecht, The APrON Team

**Affiliations:** 1 Faculty of Nursing, University of Calgary, Calgary, AB T2N 1N4 Canada; 2 Cumming School of Medicine, Departments of Pediatrics & Psychiatry, University of Calgary, Calgary, AB T2N 4N1 Canada; 3 Departments of Pediatrics, Obstetrics & Gynecology, Faculty of Medicine and Dentistry, and School of Public Health, University of Alberta, Edmonton, AB T6G 2R3 Canada; 4 Department of Psychology, University of Calgary, Calgary, AB T2N 1N4 Canada; 5 Cumming School of Medicine, Department of Pediatrics & Community Health Sciences, University of Calgary, Calgary, AB T2N 4N1 Canada; 6 Child Development Centre, ACHRI Owerko Centre, 3rd Floor, 2888 Shaganappi Trail, Calgary, AB T3B 6A8 Canada

**Keywords:** Atopic dermatitis, Childhood, Maternal–infant relationship, Sensitivity, Responsiveness, Control, Depression, Anxiety, Stress, Social support

## Abstract

**Background:**

Many studies have identified associations between qualities of maternal–child relationships and childhood asthma, but few have examined associations with childhood atopic dermatitis (AD), a common precursor to asthma. Moreover, maternal psychological distress, including prenatal and postnatal depression, anxiety and stress, may increase risk, while social support from partners may reduce risk for childhood AD. We sought to uncover the association between maternal–infant relationship qualities (maternal sensitivity towards infant behavioral signals, controlling behavior, and unresponsiveness) and child AD after accounting for risk (i.e., prenatal and postnatal maternal depression, anxiety and stress) and protective (i.e., social support) factors.

**Methods:**

We conducted a secondary analysis of data collected on a subsample of 242 women and their infants enrolled during pregnancy in the ongoing Alberta Pregnancy Outcomes and Nutrition cohort study. Inclusion criteria required mothers to be >16 years of age, English speaking and <22 weeks gestational age at enrollment. Data on depression, anxiety and stress in the prenatal and postnatal periods and physician diagnosis of childhood AD at 18 months were gathered via maternal report. Maternal sensitivity, unresponsiveness and controlling behaviours were assessed via videotaped observations using the Child-Adult Relationship Experimental (CARE)-Index at 6 months of infant age.

**Results:**

Higher maternal sensitivity, or the inability of the mother to appropriately understand and respond to infant needs based on behavioral signals, predicted reduced odds of AD independent of and in combination with low prenatal and postnatal anxiety and high paternal support. After adjustment, higher maternal controlling behaviours and unresponsiveness also predicted greater odds of AD.

**Conclusions:**

Low maternal sensitivity is a risk factor for childhood AD, independently and in combination with perinatal anxiety and low social support. Thus, interventions that improve maternal–infant relationship quality, especially sensitivity, reduce anxiety and improve social support from partners could reduce odds of childhood AD.

## Background

Atopic dermatitis (AD), a hypersensitive skin disease characterized by inflamed and scaly skin lesions, pruritus and skin rash is one of the most common chronic diseases to affect children in Western society [1]. Symptoms often appear by 6 months of age, but 65% of affected children will have symptoms by 18 months of age and in more than 50% of children, symptoms will persist through 7 years of age [2]. AD symptoms often lead to depression, sleep deprivation, feelings of embarrassment, stigma, social isolation, and restricted ability to own pets or play sports [2, 3]. Consequently, AD leads to a diminished quality of life for children [3] and its medical care burdens children, parents and the health care system [4, 5]. Substantial attention has been paid to understanding the risk factors for AD and their implications in the atopic march toward allergic rhinitis and ultimately asthma [2, 6–11]. Given that AD is a potent early warning sign for later atopic diseases, ascertaining qualities of the early environment that may predispose children to AD is warranted.

A recent systematic review identified early environmental risk factors for childhood AD to be maternal prenatal psychological distress, including depression, anxiety and stress [12]. A study of 1264 mother–infant pairs revealed that maternal report of prenatal mental status including vitality, vigor, happiness, anxiety, discouragement, nervousness, tiredness, exhaustion and work stress predicted AD in 2 year olds [13]. A large (n = 23,791) cross-sectional study revealed that parental history of diagnosed prenatal and postnatal depression predicted AD in 6–13 year olds [14]. Maternal depressive symptoms in the postnatal period were associated with AD in 3–12 month old infants as was postnatal anxiety, particularly about childrearing (n = 40) [15]. In several other studies, maternal prenatal stressful life events such as divorce, mourning the death of a loved one, job loss or financial problems were associated with AD in 3–14 year olds, independent of sociodemographic factors or known atopy risk factors, like allergen exposure [16–18]. Stressful life events during the second half of gestation were found to increase the odds of AD in 14 year olds [17]. To our knowledge studies have not examined the differential impact of prenatal or postnatal depression, anxiety and stress on the development of childhood AD.

Although prenatal and postnatal depression, anxiety and stress are often co-morbid [19–21], they likely exert unique contributions to AD development. Postnatally depressed mothers often fail to respond appropriately to infant cues, alleviate infant stress and overall, are less sensitive and responsive towards their infants [22–24]. This propensity is widely held responsible for the poor developmental outcomes often observed in children of depressed mothers [25, 26]. Prenatal psychological distress may also negatively affect the priming of the maternal brain for sensitive, responsive interactions with infants [27]. Maternal^1^ sensitivity is defined as a pattern of behaviour that pleases the infant and increases the infant’s comfort and attentiveness and reduces its distress and disengagement [28]. Consistently alleviating infant distress by responding to behavioral signals, such as fussiness due to hunger or fatigue, in a timely fashion is an indicator of high maternal sensitivity, while failing to do so is indicative of low responsiveness [29]. Moreover, high maternal sensitivity and responsiveness are contrary to maternal behaviours that are overtly or covertly hostile or attempt to overly control the infants’ behaviours in everyday interactions [30]. As a result, healthy maternal–infant relationships are typically characterized as sensitive and responsive interactions that are attentive to the infants’ needs while remaining non-intrusive and non-controlling, and contribute to the regulation of the infant’s response to stress before the infant is physiologically able to self-regulate [31–33]. In other words, infants develop their ability to regulate their stress response through their relationship with their maternal caregivers, especially during the first 3 years of life [34, 35]. Caregivers who do not provide sensitive, responsive, non-controlling care promote development of precocious self-regulation, which may have physiological costs, including suppressed immune development [36, 37].

As early as 1999, maternal–infant relationships were theorized to predict and/or compound childhood AD [38]; however, to date, only two studies have examined this theorized association [15, 39]. In contrast, many studies have identified associations between maternal–infant relationships and childhood asthma [40–45]. One study compared children with (n = 20) and without (n = 20) AD, they found that self-reported maternal overprotectiveness and control were associated with AD in 3–12 month olds [15]. Another study compared self-reported relationship characteristics of mothers of children less than 6 years of age with (n = 102) and without (n = 131) AD and found that mothers of children with AD were less affectionate and that mothers of children with more severe symptoms were more rejecting and less encouraging of children’s autonomy [39]. They also examined maternal sensitivity via parental self-report and failed to find any difference between AD and non-AD groups [39]. However, self-report measures of maternal–infant relationship quality are poorly correlated with observational measures [46] and thus observations of are much preferred [47, 48], yet much ignored in the research on AD or other atopic diseases. Given the role of AD in the atopic march and the consistent observation of associations between maternal–infant relationships and childhood asthma, and emerging evidence of associations with AD, ascertaining the influence of qualities of the maternal–child relationship (sensitivity, responsiveness, and control) using observational measures, on AD would be useful.

Furthermore, existing research has not yet examined protective factors in AD development. Social support has been described as a buffer to stressful life events, enhancing the mother’s self-esteem and self-efficacy and aiding in the transition to motherhood, consequently promoting healthy child development [49, 50]. Social support refers to emotional, affirmational, informational and instrumental assistance, from social relationships including partners [51]. Without appropriate social support during the transition to motherhood, this transition can be difficult and distressing, adding to maternal psychological distress and affecting the mother’s ability to care for her infant [49]. Lack of social support has been strongly associated with maternal depressive symptoms [51, 52]. Research has affirmed the protective role of social support, particularly from fathers, for normative and depressed mothers [53–56]. To our knowledge, existing research has not yet examined maternal social support as a protective factor in children’s AD. Other identified risk factors include parental history of atopic disease [57, 58], especially asthma, low maternal education, being a boy [59], high birth weight and day care attendance in the first year of life [60]. Finally, breastfeeding has been shown to predict both increased maternal sensitivity [53, 61] and changes in risk of AD [62].

To address the aforementioned gaps in the literature, we sought to determine the association between maternal–infant relationship qualities (sensitivity, responsiveness and control) and child AD by 2 years of age, considering risk (i.e. maternal prenatal and postnatal depression, anxiety and stress) and protective (i.e. social support) factors. Given the prominent role of maternal sensitivity, we also sought to identity predictors of maternal sensitivity. We hypothesized that lower maternal sensitivity, higher unresponsiveness and control would increase the risk for AD in young children. We also hypothesized that greater symptoms of depression and anxiety and reported stress would predict lower maternal sensitivity and increased risk of childhood AD. Finally, we hypothesized that greater reported postnatal social support would reduce the likelihood of AD.

## Methods

This is a secondary analysis of data from the Fetal Programming Study [63], a sub-study of the larger Alberta Pregnancy Outcomes and Nutrition (APrON) longitudinal pregnancy cohort study. The Fetal Programming Study was designed to examine biomarkers of maternal stress during pregnancy and to collect data on postnatal maternal–infant relationships and other health outcomes [64]. Ethics approvals were obtained from the Conjoint Health Research Ethics Board at the University of Calgary and the Health Research Ethics Board at the University of Alberta, both in Canada. After obtaining informed consent, data were collected in early (13–22 weeks) and late (32–40 weeks) gestation and again at 3 (range 2–4 months), 6 (5–8 months) and 18 (range 12–27 months) months postpartum via clinic visits and mailed questionnaires. Trained research assistants conducted clinic visits at the regional children’s hospital. No incentives to participation were offered. Details on the full cohort are published [64].

### Sample and recruitment

**Fig. 1:**
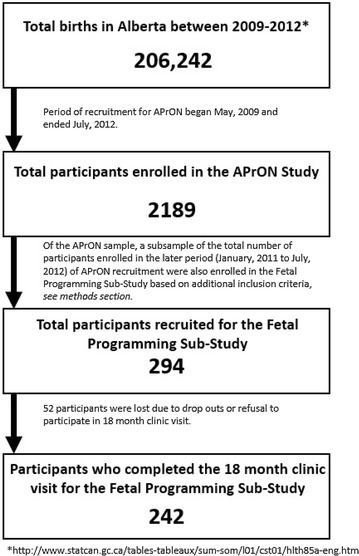
Enrollment of individuals through each stage of the study

Between 2011 and 2012, 294 participants were recruited and enrolled in the sub-study, a portion of the final total APrON cohort of 2189 mothers and their infants. See Fig. 1 for details. Due to the nature of observational measurement in the sub-sample, recruitment was constrained to Calgary. Mothers were identified via maternity, ultra sound, family medicine and obstetrics clinics and media advertisements [64]. Participant enrollment eligibility criteria included mothers <22 weeks gestational age (GA) at first study visit, >18 years of age, not smoking or drinking alcohol during pregnancy, singleton pregnancy, not on any synthetic glucocorticoid medication, and no known fetal complications during study entry [63]. At the 18 month follow-up clinic visit, the sample retained was comprised of 242 mother–infant pairs.

### Overview of study variables

This study collected data on infant AD at the 18 month clinic visit. Extensive data were collected on mothers’ demographic characteristics, health history, including maternal history of asthma, prenatal and postnatal maternal psychological distress (anxiety, depression or stress), maternal social support (specifically the extent of partner/spouse support) and children’s birth and health outcomes including breastfeeding status (at 3 months) from the APrON surveys completed at prenatal or postnatal clinic visits or sent in by mail. Observations of maternal–infant relationships were conducted at 6 month clinic visits. Covariates included identified risk factors for AD: maternal asthma, low maternal education, being a boy [59], breastfeeding status and birth weight [60]). (Another known covariate, day care attendance in the first year of life, was not included as in our Canadian sample, nearly all the children’s parents were on a 1-year parental leave.)

### Infant atopic dermatitis

This was assessed via questionnaire at the 18 month clinic visit during which mothers reported physician diagnosis of infant AD. Mothers were asked to report if their child was diagnosed by a physician with AD in the last 6 months. AD was thus dichotomized as 1 (AD diagnosis) or 0 (no AD diagnosis) for analysis. In other studies, parent report has yielded high sensitivity, specificity and concordance [65] with corroborated physician diagnosis of AD.

### Maternal–infant relationship quality

Maternal–infant relationship qualities at 6 months of age were assessed with the Child-Adult Relationship Experimental (CARE)-Index, a 5 min observational procedure involving videotaping the mother and child playing with age-appropriate, child-safe toys [28]. Video recordings were coded to attain scores for the constructs of maternal sensitivity, unresponsiveness and control. Possible scores range from 0 to 14, with higher scores indicating more observations of the construct. Moreover, sensitivity maps onto dyadic synchrony, a global measure of maternal–infant relationship quality, and scores may be categorized in the “sensitive” (11–14), “adequate” (7–10), “intervention” (5–6) and “at-risk” (0–4) ranges. The CARE-Index has a high degree of stability and has been well validated [66–71]. Typical inter-rater reliabilities range from r = 0.73–0.95 [70, 72]. Author Letourneau is reliable CARE-Index coder and supervised the administration of the measure. Video-recorded observations were coded by blinded, reliable coders who achieved 94.4% inter-rater reliability agreement on the dominant pattern (sensitivity, control and unresponsiveness). Disagreements were resolved by additional viewings and discussion.

### Prenatal and postnatal maternal psychological distress

Mothers self-reported ratings of psychological distress (depression, anxiety and stress) via the Edinburgh Depression Scale [73, 74] (EDS), Symptom Checklist-90 item-Revised (SCL-90-R) anxiety subscale [75], Pregnancy-Specific Anxiety Scale (PSAS) [76] and Stressful Life Events Questionnaire (SLEQ) [77]. All were administered in early and late pregnancy, and again at 3 months postpartum, with the exception of the administration of the PSAS only in pregnancy. The 10-item EDS has well-demonstrated reliability and validity with test–retest reliability, and good to moderate correlation with other measures of depression [73, 78, 79]. Measuring general anxiety, the SCL-90-R includes 25 items designed to reflect a symptom inventory of general anxiety [75], for which convergent and divergent validity has been established [80]. The possible range of scores for EDS and SCL-90-R is 0–30, with higher scores indicative of more severe depression or anxiety [78, 81]. For the SCL-90-R, T-scores were tabulated, with scores greater than or equal to 63 indicating high risk for anxiety. The PSAS, a 10-item self-report instrument, is a reliable measure (Cronbach’s alpha = 0.81) [54] of pregnant women’s worry regarding personal health, labor and delivery and baby care, with final scores ranging from 0 to 3. To measure the number of stressors, mothers provided self-reported ratings on the 26-item SLEQ [82, 83], indicating “yes” or “no” to whether they have experienced stressful life events, for example, a death or illness of family member or friend, divorce or separation. The range of possible scores is 0–26, with higher scores indicating a greater number of stressors. As a checklist, internal reliability or inter-item correlations are difficult to establish; however, content, construct and face validity are well recognized [82, 83].

### Maternal social support

Mothers’ perceptions of the quality of their partners’ (88% of participants reported on their child’s father) social support at 3 months postnatal was assessed via the Social Support Effectiveness Questionnaire (SSEQ), a 35-item measure of emotional/affirmational, informational, instrumental and negative support received over the previous 3 months. Total scores range from 0 to 80, with higher scores indicating more effective support from partners. The internal consistency for this instrument is strong (Cronbach’s alpha = 0.87) when used to distinguish levels of social support for childbearing women [53, 84, 85].

### Data analysis

The sample characteristics were described using descriptive summaries, including means and standard deviations. Predictor variables were dichotomized using percentiles or appropriate cut-offs (e.g. high/low or probable/not probable) to aid interpretability. To gain more insight into the relationship between maternal sensitivity and AD, maternal sensitivity was classified into the four standard categories: sensitive, adequate, intervention and at-risk, and scores ≥7 are considered “high” on sensitivity (adequate or sensitive) [28] and plotted against the percentage of children with AD. As no established cut-offs exist for maternal unresponsiveness and control, we used 75th percentile comparisons. Maximum values were used from prenatal and postnatal assessments of depression, anxiety and stress, to attain for example, the maximum depression score for the prenatal period and another maximum score for the postnatal period. These maximums were utilized in all statistical tests. Mean differences in maternal–infant relationship measures across the predictors were examined by using *t* tests and the association between AD and potential predictors was investigated using Fisher’s Chi square test. To rule out collinearity among the predictors, pairwise correlation amongst the predictors was tested using Pearson tests revealing that all the predictors were fairly or moderately (−0.35 to 0.34) correlated and suitable for inclusion in the analysis. Simple logistic regression was employed to identify potential predictors of AD. The predictors that were moderately associated (crude associations) with AD (p < 0.25) in step one and, as recommended [86], those that have previously shown to be associated with AD in other studies were selected and included in the multivariable logistic regression model. In the final analysis, two multivariable logistic models were fitted based on the study hypotheses. The first model included maternal sensitivity, maternal asthma and distress to predict AD. In the second model, maternal sensitivity was excluded and replaced with maternal unresponsiveness and control to predict AD. The goodness of fit for both models was assessed using the Pearson Chi square. Missing data were treated with listwise deletion.

## Results

In this affluent study population (Table 1), prenatal depression was found in 23.6%, prenatal anxiety in 24.1%, postnatal depression in 7.1% and postnatal anxiety in 5.6% of women. Newborns were full-term, normal weight and predominantly breastfed at 3 months. Asthma was reported in 8.3% of mothers and 18.1% of their children had AD, according to maternal report of physician diagnosis at 18 months of age.

**Table 1:** Demographic and descriptive characteristics (n = 242)

Variable	Frequency	Mean score (SD)	Percentage
*Maternal sensitivity (0–14)*			
High (≥7; adequate or sensitive)	51	7.76 (1.20)	21.3
*Maternal control (0–14)*			
High (>75th percentile)	59	8.38 (1.69)	24.7
*Maternal unresponsiveness (0–14)*			
High (>75th percentile)	56	10.4 (0.66)	23.4
*Atopic dermatitis*			
Yes	44		18.2
*Maternal asthma*			
Yes	20		8.3
*Family income*			
Above $100,000	137		58.1
*Education level*			
≥University degree	173		72.7
*Employment*			
Full time	188		80.0
*Marital status*			
Married	236		97.5
*Mother’s age*		31.2 (3.80)	
*Gestational age at birth (weeks)*		39.2 (1.70)	
*Birth weight (g)*		3355.8 (532.9)	
*Breastfeeding (3 months)*			
Yes	205		91.5
*Child sex*			
Female	120		50.6
*Pregnancy specific anxiety (0–3)*			
Probable (>75th percentile)	58	1.34 (0.25)	24.1
*Prenatal depression (0–30)*			
Probable (>9; high depressive symptoms)	57	13.2 (3.20)	23.6
*Postnatal depression (0–30)*			
Probable (>9; high depressive symptoms)	16	13.2 (2.86)	7.1
*Postnatal anxiety (T-score)*			
Probable (T-score ≥63; high risk for anxiety)	13	66.2 (2.80)	5.6
*Prenatal # of stressors*			
0	103		42.9
1	89		37.1
2	35		14.6
≥3	13		5.4
*Postnatal # of stressors*			
0	146		63.2
1	59		25.5
2	19		8.2
≥3	7		3.0
*Postnatal social support (0–80)*			
High (>50th percentile)	115	68.6 (5.40)	48.9

### Association between maternal–infant relationship quality at 6 months and child AD at 18 months

Mean scores on the CARE-Index were 5.0 (SD = 1.9) for maternal sensitivity, 6.3 (SD = 3.7) for unresponsiveness and 2.7 (SD = 3.5) for controlling in the context of maternal–infant relationships. Correlations revealed that maternal sensitivity was negatively correlated with the controlling (r = −0.19) and unresponsive (r = −0.33) domains of the CARE-Index.

Table 2 outlines descriptive and unadjusted associations among predictors, covariates and both maternal sensitivity at 6 months and AD at 18 months. Significant predictors of high maternal sensitivity (21.3% of sample) were age of women, with women over 30 years being more sensitive, and higher postnatal depressive symptoms, with the mean sensitivity score (M = 9.50, SD = 1.91) of highly symptomatic mothers being higher than the mean sensitivity score (M = 7.63, SD = 1.07) of less symptomatic mothers. The only significant predictor of AD at age 18 months (18% of sample) was maternal history of asthma.

**Table 2:** Percentage distribution of descriptive and predictor variables for mothers with high maternal sensitivity at 6 months and child AD at 18 months (Chi square tests)

	N	High maternal sensitivity at 6 months (n = 51)			Atopic dermatitis at 18 months (n = 44)		
		n	%	p value^∞^	N	%	p value^∞^
*Maternal sensitivity (0–14)*							
High (≥7; adequate or sensitive)	51				5	9.80	
Low (<7)	188				39	20.7	0.074
*Maternal asthma*							
Yes	20	3	15.0		8	40.0	
No	222	48	21.6	0.470	36	16.2	0.008^a^
*Family income*							
Below $100,000	99	22	22.2		21	21.2	
Above $100,000	137	29	21.2	0.805	23	16.8	0.389
*Education level*							
<University degree	65	10	15.4		14	21.5	
≥University degree	173	41	23.7	0.190^a^	30	17.3	0.457
*Employment*							
Part time	47	12	25.5		5	10.6	
Full time	188	39	20.7	0.962	39	20.7	0.112
*Marital status*							
Married	236				42	17.8	
Single	6				2	33.3	0.224
*Mother’s age*							
Below 30 years	70	9	12.9		16	22.8	
30 and above	169	42	24.8	0.030^a^	28	16.6	0.254
*Gestational age*							
28–38 weeks	58	14	24.1		10	17.2	
39 weeks and above	170	34	20.0	0.440	31	18.2	0.865
*Child sex*							
Male	117	21	17.9		24	20.5	
Female	120	30	25.0	0.175^a^	20	16.7	0.505
*Birth weight (g)*							
Below 2500 g	12	2	16.7		3	25.0	
2500 g and above	228	49	21.5	1.00	41	18.0	0.465
*Breastfeeding (3 months)*							
Yes	205	41	20.0	0.688	39	19.0	
No	19	5	26.3		3	15.8	0.2383
*Prenatal depression (0–30)*							
Probable (>9; high depressive symptoms)	57	9	15.8		11	19.3	
Not probable (≤9)	185	42	22.7	0.738^a^	33	17.8	0.803
*Postnatal depression (0–30)*							
Probable (>9; high depressive symptoms)	16	4	25.0		2	12.5	
Not probable (≤9)	210	43	20.5	0.003^a^	38	18.1	0.572
*Pregnancy specific anxiety (0–3*)							
Probable (>75th percentile)	58	13	22.4		14	24.1	
Not probable (≤75th percentile)	183	37	20.2	0.277^a^	30	16.4	0.183^a^
*Postnatal anxiety*							
Probable (T-score ≥63; high risk for anxiety)	13	4	30.8		4	30.8	
Not probable (T-score <63)	218	45	20.6	0.426^a^	39	17.9	0.135^a^
*Prenatal # of stressors*							
0	130	20	15.4		21	16.2	
1 or more	137	31	22.6	0.314	23	16.8	0.476
*Postnatal # stressors*							
0	146	27	18.5		28	19.2	
1 or more	85	24	28.2	0.936	15	17.6	0.773
*Maternal control (0–14)*							
High (≥75th percentile)	59				14	23.7	
Low (<75th percentile)	180				30	16.7	0.224^a^
*Maternal unresponsive (0–14*)							
High (≥75th percentile)	56				13	23.2	
Low (<75th percentile)	183				31	16.9	0.289^a^
*Postnatal social support (0–80*)							
High (≥50th percentile)	115	20	17.4		17	14.8	
Low (<50th percentile)	120	30	25.0	0.145^a^	26	21.7	0.172^a^

^∞^ p values under maternal sensitivity are based on independent t tests statistics and p values under atopic dermatitis are based on Chi square test statistics

^a^Included in models

**Fig. 2:**
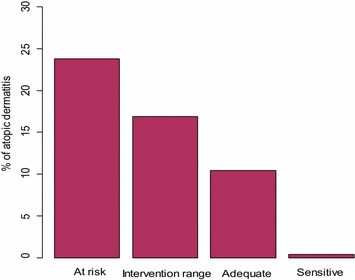
The more sensitive the mothers in the maternal–infant relationship observations, the fewer children were identified with AD. Total N = 242

Figure 2 shows that AD was reported in almost 25% of toddlers born to mothers scoring in the ‘at risk’ range of the maternal CARE-Index sensitivity measure, then dropped successively with increases to maternal sensitivity to a prevalence of less than 1% among toddlers of sensitive mothers.

### Association between maternal sensitivity, maternal distress, social support and maternal asthma and child AD at 18 months

Table 3 presents these data translated into odd ratios; maternal sensitivity was negatively associated with AD, such that this allergic skin disorder decreased in offspring by 0.81 for each unit increase in the maternal sensitivity score. Mothers with a history of asthma were also 3.4 times more likely to have a child with AD. When adjusted for maternal asthma status, prenatal or postnatal anxiety, and postnatal depression, the statistical association between maternal sensitivity and childhood AD remained; the odds ratio for AD further decreased to 0.74 for each unit increase in the maternal sensitivity score. In this model, maternal asthma and postnatal anxiety independently increased the risk for AD. The likelihood of AD was 0.73 for each unit increase in maternal sensitivity in a final model that also included postnatal social support. In this fully adjusted model, child AD was significantly more likely with prenatal or postnatal anxiety and maternal asthma status; it was inversely related to postnatal depression and postnatal social support. Neither prenatal nor postnatal stress contributed to the models.

**Table 3:** Maternal sensitivity, maternal distress, social support and maternal asthma associations with AD at 18 months

	Unadjusted OR (95% CI)	Adjusted OR (95% CI)^b^	Additional adjustment^c^
Maternal sensitivity	0.81 (0.67–0.98)^a^	0.74 (0.59–0.93)^a^	0.73 (0.56–0.93)^a^
Postnatal depression	0.97 (0.87–1.08)	0.93 (0.82–1.05)	0.86 (0.74–1.00)
Pregnancy specific anxiety	1.57 (0.76–3.27)	2.02 (0.81–5.05)	2.74 (1.04–7.19)^a^
Postnatal anxiety	1.08 (0.98–1.18)	1.13 (1.01–1.28)^a^	1.16 (1.01–1.33)^a^
Maternal asthma (ref: no)	3.44 (1.31–9.02)^a^	4.35 (1.52–12.4)^a^	5.35 (1.75–16.3)^a^
Postnatal social support	0.98 (0.96–1.01)		0.96 (0.93–0.99)^a^

^a^Significant values p < 0.05

^b^Adjusted for maternal sensitivity, postnatal depression and anxiety, pregnancy specific anxiety, maternal asthma

^c^Additional adjustment for maternal sensitivity, postnatal depression, social support and anxiety, pregnancy specific anxiety, maternal asthma

### Maternal responsiveness and control, maternal distress, maternal asthma and social support associations with AD at 18 months

**Fig. 3:**
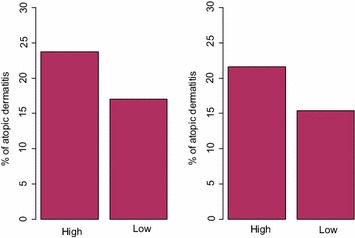
At high levels of maternal control/unresponsiveness in mother–infant relationship observations, more children have AD than children whose mothers are less controlling/more responsive. Total N = 242

Between 20 and 25% of children had AD when mothers exhibited unresponsive or controlling variants of the maternal–infant relationship, whereas they were 15% in their absence (Fig. 3). Unadjusted odds ratios for associations between unresponsiveness and controlling behaviour and AD were not significant; however, in a fully-adjusted model, independent of maternal asthma, postnatal depression or social support, child AD was 1.3–1.4 times more likely in the presence of maternal unresponsive and controlling behaviour (Table 4). As with the maternal sensitivity model, AD was independently associated with maternal asthma status, with prenatal and postnatal maternal anxiety, and inversely associated with postnatal depression and social support. Neither prenatal nor postnatal stress contributed to the models.

**Table 4:** Maternal responsiveness and control, maternal distress, maternal asthma and social support associations with AD at 18 months

	Unadjusted OR (95% CI)	Adjusted OR (95% CI)^b^	Additional adjustment^c^
Maternal unresponsiveness	1.00 (0.92–1.09)	1.31 (1.04–1.65)^a^	1.35 (1.05–1.73)^a^
Maternal controlling behaviour	1.05 (0.96–1.14)	1.32 (1.04–1.66)^a^	1.33 (1.03–1.71)^a^
Postnatal depression	0.97 (0.87–1.08)	0.93 (0.82–1.05)	0.86 (0.74–1.00)
Pregnancy specific anxiety	1.57 (0.76–3.27)	2.01 (0.80–5.06)	2.78 (1.04–7.39)^a^
Postnatal anxiety	1.08 (0.98–1.18)	1.13 (1.00–1.27)^a^	1.16 (1.01–1.33)^a^
Maternal asthma (ref: no)	3.44 (1.31–9.02)^a^	4.29 (1.49–12.29)^a^	5.39 (1.76–16.5)^a^
Postnatal social support	0.98 (0.96–1.01)		0.96 (0.93–0.99)^a^

^a^Significant values p < 0.05

^b^Adjusted for maternal unresponsiveness and controlling, postnatal depression and anxiety, pregnancy specific anxiety, maternal asthma

^c^Additional adjustment for maternal unresponsiveness and controlling, postnatal depression, social support and anxiety, pregnancy specific anxiety, maternal asthma

## Discussion

In a subsample of 242 women and their infants enrolled in the APrON longitudinal study, high maternal sensitivity was found to significantly protect against AD development at 18 months of age (adjusted OR = 0.73; 95% CI 0.56, 0.93, p = 0.012), independent of maternal asthma status. The maternal sensitivity and child AD association was also independent of maternal prenatal and postnatal anxiety, and social support. A mother’s assessment of the quality of support provided by her partner reduced the onset of this early childhood atopic disorder by 0.96 for every unit increase of perceived support. Maternal anxiety was a risk factor for AD in offspring. Further, positive associations with child AD were observed for maternal controlling and unresponsive behaviors (adjusted OR = 1.33, 95% CI 1.03, 1.71, p = 0.028 and adjusted OR = 1.35, 95% CI 1.05, 1.73, p = 0.020 respectively). Maternal sensitivity was predicted by maternal age above 30 and, in contrast to expectations, higher postnatal depressive symptoms.

Our results suggest that poor maternal–infant relationship quality (low sensitive, high unresponsive and high controlling behavior during interactions with infants) at 6 months of age increases the risk of child AD at 18 months of age. Our findings are consistent with evidence demonstrating the link between maternal–infant relationship quality and child atopic disease, although current understanding is largely limited to the etiology of asthma [40–43, 45]. For example, identified difficulties in the parent–child relationship independently predict asthma onset at 3 and 6 years of age [41], while the onset of asthma in early life has been associated with poor quality relationships with parents that persist into adulthood [87].

Infants whose caregivers are less sensitive have higher levels of cortisol [33, 88] and excess cortisol exposure during early infancy may influence the developing immune response via epigenetics or neuroendocrine dysregulation, potentially leading to AD [87, 89, 90]. It is also possible that more sensitive mothers take greater care of their infants’ skin, by for example, use of moisturizer and regular bathing; however, how this may contribute to the atopic march toward asthma is unclear. To our knowledge, no evidence has been published demonstrating associations among maternal sensitivity, infant skin care, and the atopic march, but these associations are worth exploration as possible rival hypotheses. A recent review summarizing the increasing literature on links between maternal–infant relationships and asthma development, noted that only correlational, not causal links have been established. The review called for randomized controlled trials of social interventions focused on maternal–infant relationships with examination of atopic disease outcomes [44].

Predictors of maternal sensitivity typically include low postpartum depression, anxiety, and stress, high paternal support and adult (as opposed to adolescent) motherhood [91–93]. Depression is known to deplete maternal ability to express positive affect towards her infant, and could possibly be exacerbated by lack of paternal support [91]. Our finding that older adult mothers are more sensitive to their infants is in keeping with research suggesting that younger mothers engage less frequently than older adult mothers in behaviors that promote secure attachment, such as positive verbal feedback [94]. But, contrary to much evidence [61, 62, 90, 92, 93, 95], neither maternal stress, anxiety nor breastfeeding were significantly associated with maternal sensitivity. We also observed an unexpected association between higher postnatal depressive symptoms and maternal sensitivity, which may have been due to small sample size. Women reporting excessive depression, anxiety or stress are typically less positively reinforcing and interactive with their infant, thought to lead to poor child development outcomes [22–24, 96]. However, new data from the Avon Longitudinal Study of Parents and Children (n = 704) revealed that perinatal depressive symptoms predicted little variance in maternal–infant relationship qualities, but rather maternal interpersonal sensitivity in pregnancy is the most significant contributor to forming healthy maternal–infant relationships in the context of perinatal depressive symptoms [97]. In other words, other variables that are correlated with both depressive symptoms and qualities of the maternal–child relationship may underpin observed associations between depression and child development and deserve exploration. Moreover, depressive and anxiety symptoms in pregnancy are typically worse than in the postpartum period [98, 99], so significant differences would be expected; however, how these differences may influence maternal–child relationships or onset of children’s AD is unknown. Studies have shown that high levels of perceived support reported by depressed or anxious mothers may buffer the association with child atopic disease, suggesting accessible support may improve the quality of the mother–child relationship [100–102]. In this study, although greater reported postnatal social support was shown to reduce the likelihood of AD, consistent with various studies [34, 53, 54], social support was not found to be a predictor of sensitive maternal behavior.

Prenatal pregnancy-specific anxiety (adjusted OR = 2.74; 95% CI 1.04, 7.19, p = 0.041) and postnatal anxiety (adjusted OR = 1.16, 95% CI 1.01, 1.33, p = 0.034) predicted AD independent of paternal support and maternal sensitivity. A trend for an inverse association between postnatal depression and AD was also observed. These findings are compatible with several studies on childhood atopic disease associations with maternal prenatal or postnatal stressors [13, 16–18, 59, 103], prenatal anxiety but not depression, as suggested above [88, 104–107]. Mutual adjustment for anxiety and depression may have uncovered the negative influence of postnatal depression on the health care utilization for infant care and thus, physician diagnosis of AD [108]. Consistent with our results, some studies found postnatal anxiety about childrearing to be associated with AD in 3–12 month old infants [15]. As maternal anxiety becomes more prevalent [109], it may gain a more prominent influence on the maternal–infant relationship [97]. Given that low maternal sensitivity and anxiety are interrelated, the association with childhood AD may be similarly mediated by epigenetics or neuroendocrine dysregulation [88, 104, 110], and altered infant cytokine profiles [111]. Infant neuroendocrine and immunologic maturation is highly plastic in the face of environmental stressors [112–115], especially during the early postnatal period. For those who are already genetically susceptible to the development of atopy [116], as observed in our sample of children whose mothers reported a history of asthma, maternal sensitivity may make the difference between the development of AD or not.

The pathogenesis of AD is known to involve abnormal levels of specific cytokines released by T-helper 2 cells detected in cord blood or infant peripheral blood [117–120]. The altered differentiation of these cytokines may be induced through excessive glucocorticoid exposure at an early age, driven by maternal perinatal psychosocial distress [121, 122]. Future research could focus on understanding the associations among: (1) excessive, long-term fetal/infant glucocorticoid exposure during critical developmental periods, (2) Th2 specific cytokine levels in infant cord or peripheral blood, and (3) fetal programming, driven by prenatal distress (anxiety, depression and stress), and (4) poor maternal–infant relationship qualities, linked to postnatal distress. Moreover, genetic risk, suggested by the heritability of atopic disease suggest a need for genome-wide association studies [13].

This study has many strengths, including the outcome of childhood AD assessed via maternal report of a physician diagnosis of AD and observational assessment of maternal–infant relationship quality, but there are also limitations. First, physician corroboration of maternal report of physician diagnosis of AD was not possible. Second, only “maternal” caregiving was assessed; rather than seeking to reinforce gender stereotypes, we recognize that primary caregivers may be mothers, fathers or others. However, we also recognize that the vast majority of infants’ primary caregivers are mothers in Canada [123] and in our study [64]. Third, only a small number of mothers reported depressive symptoms in the postpartum period. This may have contributed to the potentially spurious findings of inverse association between postpartum depression and outcomes. Additionally, not all participants reported perceptions of the quality of their partners’ support, as only 88% of participants reported on their child’s father. Nonetheless, the variability in primary support provider may also reflect the reality of modern families. Furthermore, while we collected data on maternal asthma, we did not collect additional data on mother’s atopy status or family history of atopy. Finally, we acknowledge that this is a relatively high SES sample with low sociodemographic risk that may limit generalizability.

To our knowledge, this is the first study to uncover the association between maternal–infant relationship qualities (sensitivity, control, unresponsiveness) and childhood AD after accounting for risk (maternal depression, anxiety and stress) and protective (social support) factors and well-known covariates. We found low maternal sensitivity to be a significant risk factor for childhood AD, in the presence and absence of perinatal anxiety and low social support. Caregivers who are emotionally engaged and supportive of their children during infancy may be well equipped to prevent poor infant immunological development, reducing the likelihood of childhood AD and potentially asthma [34]. Limited work has begun to establish that interventions focused on the quality of maternal child-relationship may influence asthma symptoms [45]. These results suggest interventions that improve maternal–infant relationship quality, anxiety and support could reduce odds of childhood AD. Existing parenting intervention programs, including the Nurse Family Partnership [124] and Keys to Caregiving [125], and support services may already have effects on AD and the atopic march, that have not to date been measured. In addition, strengthening of relationships between caregivers serves as a means of controlling maternal anxiety [95] and may therefore be effective in helping prevent childhood AD. Additional intervention studies ought to focus on improving maternal child relationship quality and reducing odds of AD, as a means to forestall the atopic march to allergic rhinitis and ultimately asthma.

## Conclusions

High maternal sensitivity and social support, in combination with low perinatal anxiety, are associated with reduced odds of childhood AD. Higher maternal control and unresponsiveness are associated with increased odds of childhood AD. Clinical assessment of perinatal anxiety, improved social support and the introduction of early intervention programs designed to improve maternal–infant relationship quality may reduce the odds of childhood atopic dermatitis and the progression towards the atopic march, as well as help uncover genetic factors and explanatory neuroendocrine and epigenetic mechanisms.

## Abbreviations

AD: atopic dermatitis
APrON: Alberta Pregnancy Outcomes and Nutrition
GA: gestational age
CARE-Index: Child-Adult Relationship Experimental-Index
EDS: Edinburgh Depression Scale
SCL-90-R: Symptom Checklist-90 item-Revised
PSAS: Pregnancy-Specific Anxiety Scale
SLEQ: Stressful Life Events Questionnaire
SSEQ: Social Support Effectiveness Questionnaire

## Footnotes

1 We recognize that mothers are not the only caregivers for children and that relationships with primary caregivers and children are paramount, regardless of gender; however, all research to date and reviewed here has focused on mothers.
